# Bilateral somatosensory evoked potentials following intermittent theta-burst repetitive transcranial magnetic stimulation

**DOI:** 10.1186/1471-2202-11-91

**Published:** 2010-08-05

**Authors:** Azra Premji, Angela Ziluk, Aimee J Nelson

**Affiliations:** 1 Department of Kinesiology, University of Waterloo, Waterloo, Canada

## Abstract

**Background:**

Intermittent theta-burst stimulation (iTBS) is a form of repetitive transcranial magnetic stimulation that may alter cortical excitability in the primary somatosensory cortex (SI). The present study investigated the effects of iTBS on subcortical and early cortical somatosensory evoked potentials (SEPs) recorded over left, iTBS stimulated SI and the right-hemisphere non-stimulated SI. SEPs were recorded before and at 5, 15, and 25 minutes following iTBS.

**Results:**

Compared to pre-iTBS, the amplitude of cortical potential N20/P25 was significantly increased for 5 minutes from non-stimulated SI and for 15 to 25 minutes from stimulated SI. Subcortical potentials recorded bilaterally remained unaltered following iTBS.

**Conclusion:**

We conclude that iTBS increases the cortical excitability of SI bilaterally and does not alter thalamocortical afferent input to SI. ITBS may provide one avenue to induce cortical plasticity in the somatosensory cortex.

## Background

Repetitive transcranial magnetic stimulation (rTMS) over primary somatosensory cortex (SI) may alter touch sensation and physiology. At high and low frequencies, rTMS may improve [1,2] or impair [3,4] touch perception, respectively, and alter the amplitude of somatosensory evoked potentials (SEPs) [5]. One rTMS approach coined theta-burst stimulation (TBS) delivers high-frequency TMS bursts at a low intensity for a short duration, and may alter cortical excitability for up to 60 minutes [6]. TBS delivered continuously (cTBS) or intermittently (iTBS) results in inhibition or excitation of the cortex, respectively (for review see ref [7]). When applied over the primary motor cortex (M1), cTBS decreases motor evoked potentials (MEPs) [6,8-10] while iTBS increases MEP amplitude [6,8,10].

Few investigations have examined the effects of TBS over SI. Reports indicate that cTBS decreases [11] and iTBS increases [12,13] the amplitude of cortical SEPs recorded ipsilateral to TBS stimulation. However, it remains unclear whether SEPs recorded over SI contralateral to iTBS stimulation may be modulated. Such alterations were not observed using paired-pulse SEP suppression [13], however, recent reports implore the use of corrective adaptation to calculate true suppression [14,15], and the origin of the suppressive effect itself, although calculated from cortical SEP components, may occur at any synapse rostral to the brainstem [16]. Conventional SEP methods that involve averaging evoked potentials to epochs of single stimuli do not suffer from ambiguities surrounding their origin and interpretation [17]. Further, single versus paired-pulse techniques may yield results that differ and reflect subtleties associated with one or the other method [18]. Using single pulse SEPs, cTBS had no significant effect on SEPs recorded from non-stimulated SI [11]. The impact of iTBS on SEPs recorded from the non-stimulated SI has yet to be investigated and alterations would support the functional connectivity between homologous SI areas as reported elsewhere [19,20]. The present study investigates the effects of iTBS on cortical SEPs recorded from the stimulated and non-stimulated hemisphere.

The effect of TBS on subcortical SEPs remains unclear. SEP component P18 reflects neural impulses traversing thalamocortical afferents [21], and TBS-induced alterations in this potential may be the source of subsequent changes in cortex. Following cTBS to left-hemisphere SI, subcortical potentials recorded from bilateral SI were not altered [11]. The effects of iTBS on subcortical potentials have not, to our knowledge, been investigated. We address this question by examining the effects of iTBS on subcortical SEPs recorded from SI bilaterally. Identifying such modulation would yield an opportunity to induce excitability changes in subcortical synapses that in turn, may impact a large cortical territory due to their divergent terminations within cortex.

The present study examined the effects of iTBS on SI and thalamocortical physiology in both hemispheres. Specifically, we sought to determine whether iTBS would alter subcortical and/or cortical SEPs evoked from the stimulated and non-stimulated hemisphere. We recorded subcortical and early cortical SEPs before and after iTBS applied to left-hemisphere SI. SEPs were recorded from iTBS stimulated SI and right-hemisphere non-stimulated SI. We hypothesized that iTBS would increase the peak-to-peak amplitude of early cortical SEPs (N20/P25) in the stimulated SI [12,13]. Based on growing evidence that callosal connections exist between SI cortices in monkeys and humans [22-24] and SEPs are modulated by contralateral TMS conditioning [25], we also hypothesized an increase in cortical SEPs in the non-stimulated SI. ITBS induced effects may be due to changes in subcortical thalamocortical afferent input, and we therefore hypothesized that subcortical potential P18/N20 would increase bilaterally following iTBS.

## Results

### Left-hemisphere SEPs (stimulated SI)

**Figure 1:**
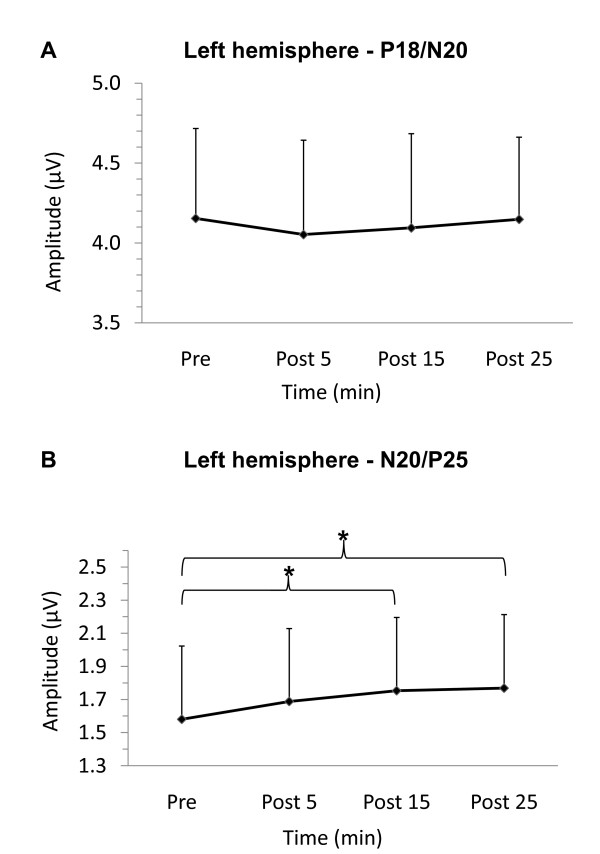
**Left-hemisphere SEPs**. Group averaged SEPs recorded from iTBS stimulated left-hemisphere SI. A. P18/N20 peak-to-peak group averaged data (n = 11) before (pre) and following (post) iTBS at 5 min (post 5) 15 min (post 15) and 25 minutes (post 25). B. N20/P25 peak-to-peak group averaged data pre and post-iTBS (n = 11). SEP amplitude is significantly greater at 15 and 25 min following iTBS (p < 0.05). Error bars represent standard error of the mean.

For the P18/N20 potential, there was no significant main effect of TIME (F_(3, 30) _= 0.19, p = 0.90) and SEP amplitudes were not significantly different following iTBS. Figure 1A plots the group averaged peak-to-peak amplitude (n = 11) of the subcortical P18/N20 SEP before and after iTBS. For the N20/P25 peak-to-peak amplitude, ANOVA revealed a main effect of TIME (F_(3, 30) _= 3.38, p = 0.03). Figure 1B plots the group averaged peak-to-peak N20/P25 amplitude (n = 11). Compared to pre-iTBS, the N20/P25 was significantly greater at 15 (p = 0.013) and 25 minutes post-iTBS (p = 0.007).

### Right-hemisphere SEPs (non-stimulated SI)

**Figure 2:**
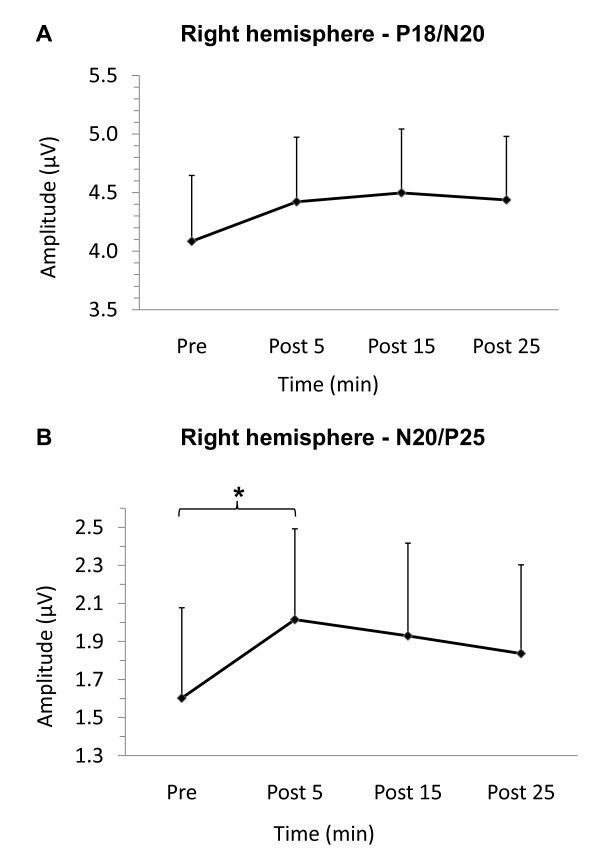
**Right-hemiphere SEPs. Group averaged SEPs recorded from right-hemisphere, non-stimulated SI**. A. P18/N20 peak-to-peak group averaged data (n = 11) pre and post-iTBS. B. N20/P25 peak-to-peak group averaged data (n = 11) pre and post-iTBS. SEP amplitude is significantly greater at 5 min following iTBS only (p < 0.017, Bonferroni correction for 3 comparisons). Error bars represent standard error of the mean.

Following iTBS over the left-hemisphere, the subcortical P18/N20 SEP was not significantly altered (TIME (F_(3, 30) _= 1.08, p = 0.37). Figure 2A plots the group averaged peak-to-peak amplitude (n = 11) of the subcortical P18/N20 SEP. The ANOVA testing the N20/P25 peak-to-peak amplitude revealed a near significant effect of TIME (F_(3, 30) _= 2.48, p = 0.08). The a priori contrasts comparing pre- and post-iTBS SEPs revealed that the N20/P25 peak-to-peak amplitude was significantly greater at 5 (p = 0.015) but not 15 (p = 0.049) or 25 minutes (p = 0.153) following iTBS. Figure 2B plots the group averaged peak-to-peak N20/P25 data.

## Discussion and Conclusion

The present study revealed an increase in the cortical N20/P25 SEP component in the iTBS stimulated SI in support of previous findings [12,13]. Novel findings include the observation that this potential was also modulated in the non-stimulated SI immediately following iTBS, and that subcortical SEPs were unaltered bilaterally. These results suggest that iTBS facilitates SEPs via an increase in the excitability of SI cortex bilaterally.

The N20/P25 potential is considered to be of cortical origin whereby the N20 potential reflects layer 4 depolarization in Brodmann area 3b and the P25 is generated by the depolarization of apical dendrites located in cortical layers 2/3 [17]. Increases in the N20/P25 recorded from stimulated SI were not observed immediately following iTBS but rather at 15 and continued for at least 25 minutes, similar to that reported elsewhere [12]. The delayed and longer-lasting facilitation observed following iTBS is in contrast to the immediate suppressive and short-lasting effects on cortical SEPs after cTBS to SI [11]. Further, the magnitude of SEP facilitation we observed (approximately 0.2 μv) is similar to that previously reported for iTBS to SI [12] and is slightly less than the SEP attenuation that follows cTBS to SI [11]. An explanation for the differences observed in the onset, duration and magnitude of the iTBS versus cTBS when applied to SI are not presently known. However, when delivered over M1, cTBS alters early I-waves [26] and iTBS affects later I-waves [27], suggesting that the TBS protocol selectively alters specific populations of interneurons. The same may be true for cTBS and iTBS to SI but this remains to be investigated.

The N20/P25 SEP recorded over the non-stimulated, right-hemisphere SI was increased following iTBS for a short duration. As proposed elsewhere, SI may exert a net inhibitory influence on the homologous SI [28] with mechanisms similar to that which mediate interhemispheric inhibition [29-31]. If true, one might expect iTBS to facilitate the excitatory transcallosal neurons that in turn synapse on local inhibitory interneurons within contralateral SI, thus leading to increased inhibition within the non-stimulated SI. Mochizuki et al. (2007) report decreased oxy-Hb in left SI following iTBS to right SI [28]. Further, fMRI activity in left SI is decreased during 10 Hz TMS bursts to right parietal cortex [32]. At first glance these findings appear to contradict the increased SEPs in non-stimulated SI in the present study. However, the latter findings of decreased SI responses were only observed in the absence of peripheral input. Blankenburg et al. (2008) further report that fMRI activity in left SI increased following rTMS to right parietal cortex when TMS was combined with median nerve stimulation [32]. Similarly, we observed an increase in stimulus-evoked activity in non-stimulated SI, but have not examined the ongoing cortical activity, which we predict would be decreased (i.e. increased inhibition) as has been shown using neuroimaging [28,32]. Therefore, we speculate that iTBS effects on the non-stimulated SI act to facilitate the stimulus-evoked response and increase the baseline inhibition. It remains unclear why the facilitation of SEPs are short lasting but it is notable that the cessation of this latter effect coincides with the onset of the facilitation of SEPs in stimulated SI (~15 minutes). The suggestion that homologous SI loci are mutually inhibitory lends support to our speculation [28]. It is also noteworthy that immediately following rTMS to right M1, SEPs in left SI are increased while those from the rTMS stimulated hemisphere were unchanged [33]. In contrast to our findings, Ragert et al. (2008) found no changes in non-stimulated SI following iTBS as measured via paired-pulse SEPs, an effect that may reflect differing methodologies [13]. Conditioning TMS pulses to the right-hemisphere altered SEPs recorded from the left-hemisphere when evoked by conventional single pulses but not paired-pulse stimulation [34].

ITBS did not modulate subcortical potentials when delivered at 80% AMT intensity. The SEP component P18 is typically recorded from C3'/C4' electrodes referenced to Fz, and is believed to reflect thalamocortical afferent transmission [21]. Alternative protocols applied over SI such as paired associative stimulation and cTBS have similarly reported a lack of change in the amplitude of subcortical potentials following stimulation [11,35] suggesting that rTMS effects may be limited to cortical rather than subcortical structures. Collectively, the P18/N20 and N20/P25 data suggest that the effects of iTBS target the upper cortical layers where early cortical potentials are generated. It may be that iTBS delivered at greater stimulator intensity will modulate subcortical potentials.

ITBS to SI may have different effects on SEPs generated in SI versus evoked potentials generated in remote areas. In the present study, iTBS was applied to SI and SEPs components generated within the stimulated and non-stimulated SI were increased. In contrast, a study using laser evoked potentials demonstrated that iTBS applied to SI decreased potentials generated within the ipsilateral SII region and had no effect on potentials from the SII contralateral to iTBS [36]. Another possibility is that compared to median nerve evoked SEPs, laser evoked potentials activate different afferent types and a smaller population of afferents conveyed through different ascending paths to distinct regions within the thalamus and cortex [37]. Any of these differences may alter the sensitivity of the resulting laser evoked potential to iTBS induced changes.

Some methodological factors require consideration and may influence the interpretation of the present results. First, similar to previous studies examining the influence of TBS on SEP amplitude [11-13], we did not include a TBS sham control group and this would be useful in ruling out placebo effects. Second, the effects observed may relate specifically to the intensity and direction of iTBS current. ITBS was delivered at 80% AMT with the induced current flowing in the posterior to anterior direction within the cortex. CTBS effects may be specific to current direction [8,38] but these findings are not unanimous [10]. It is even less clear that iTBS effects are determined by the direction of induced current [38]. Further, it is not known whether iTBS effects are directed at ongoing cortical activity or whether the effects are specific to the population of neurons responsive to the peripheral input. Further studies using multiple electrode locations and spectral analysis during pre-stimulus versus stimulus periods may address this question.

The present study found that increasing the excitability in SI via iTBS increases the amplitude of cortical SEPs in both the stimulated and non-stimulated SI. Therefore, iTBS, delivered using the published protocol [6] may provide one avenue to promote plasticity in the somatosensory cortex not only at the site of stimulation but also within the contralateral somatosensory cortex. The effects observed were restricted to cortical potentials and iTBS did not exhibit any effect on subcortical SEPs. Further studies are needed to investigate if the magnitude and duration of iTBS effects accumulate over repeat sessions, and whether perceptual changes are observed on the hand ipsilateral to iTBS.

## Methods

### Participants

Eleven right-handed subjects (7 females, aged 22-36, mean age 26 ± 4.25 years) participated. Right-handedness was determined using the Waterloo Handedness Questionnaire (WHQ). All subjects gave informed written consent. This study was approved by the Office of Research Ethics at the University of Waterloo and conformed to the Declaration of Helsinki.

### Somatosensory Evoked Potentials

SEPs were evoked by electrical stimulation of the right and left median nerves at the wrist (Grass SD9 Telefactor stimulator) using a square wave pulse of 200 μs delivered at 3 Hz at an intensity of 2.5 times sensory threshold, a stimulus intensity that elicits SEPs of submaximal amplitudes [39] but sufficient to cause a twitch in the thenar muscles in all subjects. Median nerve bar electrodes were held in place using a velcro strap with cathode proximal. Ag-AgCl scalp electrodes were placed at C3' (2 cm posterior to C3) and C4' (2 cm posterior to C4), and referenced frontocentrally to Fz according to the International 10-20 system [40]. FMRI data suggest that C3' overlays the left-hemisphere somatosensory area representing the hand [41]. Further, previous iTBS studies used these montages for SEP recordings [12,13,35,41]. A ground electrode (Ag-AgCl) was placed on the clavicle. Signal software (Cambridge Electronic Design Ltd.) was used to deliver the electrical stimulus and collect SEP data. SEPs were sampled at 5 kHz using a 10 K gain and filtered between 1 and 2500 Hz. To ensure that the same scalp electrode recording positions were used before and after iTBS, the C3' left-hemisphere SI electrode position was marked on the scalp, removed for iTBS delivery and subsequently replaced as done elsewhere [13]. Electrode impedance was maintained below 5 kΩ and checked at several intervals before and after iTBS delivery using the Mastercraft Digital Multimeter (Colluck Company Ltd). SEPs were recorded in blocks of 500 epochs immediately before iTBS and at 5, 15 and 25 minutes after the cessation of iTBS. The order of right versus left median nerve stimulation was randomized across participants.

### EMG recording

Surface EMG was recorded from the right first dorsal interosseous (FDI) muscle with 9 mm diameter Ag-AgCl surface electrodes. The active electrode was placed over the muscle belly and the reference electrode was placed over the metacarpophalangeal joint of the index finger. EMG was amplified 1000 ×, band-pass filtered between 2 Hz to 2.5 kHz (Intronix Technologies Corporation Model 2024F, Bolton, Ontario, Canada), digitized at 5 kHz by an analog-to-digital interface (Micro1401, Cambridge Electronics Design, Cambridge, UK) and stored on a computer for off-line analysis.

### Neuronavigation and Transcranial magnetic stimulation

**Figure 3:**
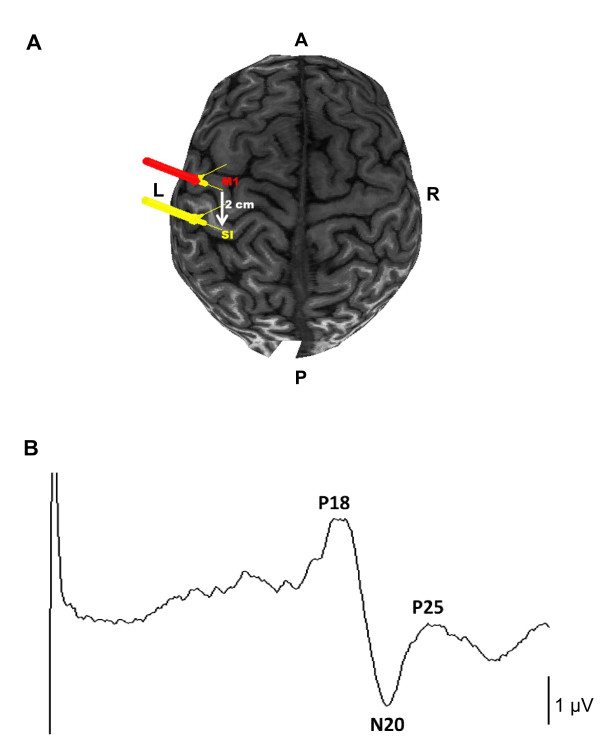
**A. Using Brainsight neuronavigation and the MRI for each individual left SI was marked 2 cm posterior to the motor representation of the right FDI muscle within M1**. A (anterior), P (posterior), L (left-hemisphere), R (right-hemisphere), M1 (primary motor cortex), SI (primary somatosensory cortex). B. Subject trace following right median nerve stimulation (average of 500 stimulation epochs) and recorded from C3' - Fz. Components of the MN-SEP: P18, N20, P25.

TMS was performed using a 90 mm outer diameter figure of eight coil with a MagPro stimulator (MCF-B65; Medtronic, Minneapolis, MN, USA). Brainsight Neuronavigation (Rogue Research, Montreal) was used to monitor the position of the TMS coil. The motor hotspot was defined as the M1 location optimal for eliciting a MEP in the contralateral FDI muscle with the coil oriented 45 degrees to the mid-sagittal line. Active motor threshold (AMT) was determined at this location and defined as the lowest intensity required to evoke MEPs of >200 μV amplitude in 5 out of 10 consecutive trials during 10% of the maximum voluntary contraction (MVC) of FDI [42]. MVC was determined by having each participant abduct their right FDI against the base of the Brainsight apparatus. The EMG signal was passed through a leaky integrator and displayed as a bright line on an oscilloscope whereby the line position reflected the level of EMG. After determining MVC, the experimenter positioned a second line on the oscilloscope corresponding to 10% of the MVC. The subject was required to position the EMG controlled line over the line marking the 10% MVC. ITBS was applied at an intensity of 80% AMT using the published protocol [6,12,13] with the handle pointing backward and laterally at a 45 degree angle away from the midline to induce current flowing from posterior to anterior within the cortex. The average AMT used in the study was 49.38% (S.D. ± 8.7) of the stimulator output. ITBS was performed over SI, defined as a point 2 cm posterior from the motor hotspot, a position that overlies the postcentral gyrus [43]. This position corresponds closely to C3'[35] and rTMS protocols applied over this location have modulated the amplitude of cortical SEPs recorded from this electrode [11,35]. The position of the coil over the postcentral gyrus was confirmed for each subject using his or her MRI combined with Brainsight Neuronavigation. MRI was conducted on a 3T GE scanner (172 images) with 3DFSPGR-IR sequences using a 20 cm FOV (256 × 256). Figure 3A displays an example of the location of the iTBS target for one participant.

### Data Analysis

Trials exhibiting movement or noise artefacts were rejected on-line and also during the post collection analysis. The peak-to-peak SEP amplitude was measured for the P18/N20 subcortical and the N20/P25 cortical potential by averaging 500 stimulation epochs for each nerve stimulated. Figure 3B displays an example of a SEP average trace (n = 500) for one participant. Four one-way repeated measures analyses of variance (ANOVA) were performed (right-hemisphere P18/N20, right-hemisphere N20/P25, left-hemisphere P18/N20 and left-hemisphere N20/P25) using within-subject factor TIME (4 levels; pre, post 5 min, post 15 min, post 25 min). Statistical analysis was performed with SAS 9.1.3. Windows software (SAS inc., Cary, North Carolina, US). A priori contrasts tested the hypotheses that subcortical P18/N20 and cortical N20/P25 SEPs are greater at 5, 15 and 25 minutes following iTBS. Post-hoc analysis was performed using Dunnett's test. Significance was set at p ≤ 0.05.

## Competing interests

Dr. A. Nelson received research grants from the Dystonia Medical Research Foundation, infrastructure grants from the Canada Foundation for Innovation and an operating grant from the Natural Science and Engineering Research Council of Canada.

Ms. Premji and Ms. Ziluk declare that they have no competing interests.

## Authors' contributions

AP conceived of the study, carried out data collection, data analysis, and writing the manuscript. AZ assisted with data collection and writing the manuscript. AN conceived of the study, carried out data analysis and interpretation and assisted with writing the manuscript. All authors read and approved the final manuscript.
